# Exploring cutaneous lymphoproliferative disorders in the wake of COVID‐19 vaccination

**DOI:** 10.1002/ski2.367

**Published:** 2024-03-25

**Authors:** Emily R. Gordon, Oluwaseyi Adeuyan, Bradley D. Kwinta, Celine M. Schreidah, Lauren M. Fahmy, Dawn Queen, Megan H. Trager, Cynthia M. Magro, Larisa J. Geskin

**Affiliations:** ^1^ Vagelos College of Physicians & Surgeons Columbia University New York NY USA; ^2^ Department of Dermatology Columbia University Irving Medical Center New York NY USA; ^3^ Department of Pathology and Laboratory Medicine Weill Cornell Medicine New York NY USA

## Abstract

**Background:**

Individual reports have described lymphoproliferative disorders (LPDs) and cutaneous lymphomas emerging after administration of the COVID‐19 vaccine; however, the relationship between reactions and vaccine types has not yet been examined.

**Objective:**

Determine if there are cases of cutaneous LPDs associated with certain COVID‐19 vaccines and their outcomes.

**Methods:**

We analysed PubMed, the Vaccine Adverse Events Reporting System (VAERS), and our database for instances of biopsy‐proven LPDs following COVID‐19 vaccines.

**Results:**

Fifty cases of biopsy‐proven LPDs arising after COVID‐19 vaccination were found: 37 from medical literature, 11 from VAERS and two from our institution. Geographical distribution revealed the most cases in the United States, Italy, and Greece, with single cases in Spain, Colombia, Canada, Japan, and Romania. The average age of patients was 53; with a slight male predominance (male‐to‐female ratio of 1.5:1). The Pfizer‐BioNTech vaccine was associated with LPDs in 36/50 (72%) cases, aligning with its 70% share of the global vaccine market. Histopathology revealed CD30+ in 80% of cases. The most prevalent form of LPD was lymphomatoid papulosis (LyP, 30%). All reported cases produced favourable outcomes (either complete or near‐complete remission). Therapeutic approaches ranged from observation to treatment with steroids, methotrexate, or excision.

**Conclusion:**

LPDs after COVID‐19 vaccination appear in the context of the same vaccines (proportionally to their global market shares), share clinical and pathological findings, and have indolent, self‐limited character.

## Graphical abstract

Reports describe lymphoproliferative disorders after COVID‐19 vaccination. We analyse world literature to report on remarkable consistencies such as reactions to the same vaccines and shared clinical and pathological findings. COVID‐19 vaccination may be associated with reactive and indolent lymphoproliferative disorders, thus unusual reactions merit diagnostic biopsies. Reactions occur based on vaccine type proportionally to global market share.

**What is already known about this topic?**

- Reports describe lymphoproliferative disorders after COVID‐19 vaccination.

**What does this study add?**

- We analyse world literature to report on remarkable consistencies such as reactions to the same vaccines and shared clinical and pathological findings. COVID‐19 vaccination may be associated with reactive and indolent lymphoproliferative disorders, thus unusual reactions merit diagnostic biopsies. Reactions occur based on vaccine type proportionally to global market share.

## 1 INTRODUCTION

The first COVID‐19 vaccines were released in December 2020. Vaccines have been produced by Pfizer‐BioNTech, Moderna, Oxford‐AstraZeneca, and Johnson & Johnson, among others. Cutaneous reactions after COVID‐19 vaccination have included local injection site reactions and cutaneous hypersensitivity reactions reflective of adaptive immune responses to antigenic components in the vaccine or unmasked hypersensitivity to non‐vaccine antigens.^1^ Primary cutaneous lymphoproliferative disorders (LPDs) after vaccination are reported but uncommon.

We provided an in‐depth analysis of 50 LPDs after COVID‐19 vaccination.^2^ While it is important to consider the reactions, it is also essential to investigate the vaccines themselves, elucidating their similarities and differences. Here, we analyse the nature of these vaccines, exploring the types of reactions associated with vaccine subtypes, their demographic distribution, and their mechanisms.

## 2 METHODS

We searched PubMed, reviewed the Vaccine Adverse Events Reporting System (VAERS), and queried our institutional database, for LPDs following COVID‐19 vaccination. We searched PubMed from December 2020 through September 2023 for ^“^COVID‐19 vaccination” and “lymphoproliferative disorder” or “cutaneous lymphoma” followed by specific searches for subtypes such as “lymphomatoid papulosis” or “anaplastic large cell lymphoma” along with others. All COVID‐19 vaccine types were included. We similarly searched VAERS for reactions, starting broadly with lymphoproliferative disorders and narrowing to specific subtypes.^3^ We reviewed the reported cases for clinical and histopathologic descriptions and cross‐referenced between the medical literature and VAERS to ensure cases were not counted twice. Cases lacking biopsy proven LPDs were excluded. We used Our World in Data, an epidemiological tool for tracking COVID‐19 vaccination rates, to assess relative distributions of vaccines by country.^4^ For each vaccine (e.g., Pfizer‐BioNTech), we investigated rates and types of reactions, gender, mean age, country, and histopathology.

## 3 RESULTS

In our updated review of the literature through September 2023, 55 cases of LPDs after COVID‐19 vaccination were identified: 42 from the medical literature, 11 from VAERS, and two from our institution. We included 50 of 55 cases in our analysis, excluding five due to incomplete demographic and histologic data.

### 3.1 LPD types

Forty‐one new onset LPDs after COVID‐19 vaccination were documented including pityriasis lichenoides et varioliformis acuta (PLEVA), T‐cell predominant cutaneous lymphoid hyperplasia (CLH), primary CD30+ LPD, lymphomatoid papulosis, subcutaneous panniculitis‐like T‐cell lymphoma (SPTCL), and primary peripheral T‐cell lymphoma.^[^ ^2^, ^5^, ^6^, ^7^, ^8^, ^9^, ^10^, ^11^, ^12^ ^]^ Twenty‐nine of 41 (71%) new LPDs occurred after the Pfizer‐BioNTech vaccine, 7 after Moderna, 4 after Oxford‐AstraZeneca, and 1 after Johnson & Johnson. Twenty‐three of 41 (56%) new LPDs presented in men, with 18 in women. Mean age of new LPDs was 52 years (range 15–80 years). For geography, 23 of 41 (56%) occurred in the US, 11 in Italy, 2 in Greece, and 1 in Colombia, Spain, Canada, Romania, and Japan. Timing of new LPD onset after vaccination was 13 days (range 2–28 days).

Nine cases of relapses of LPDs were reported including primary cutaneous anaplastic large cell lymphoma (pcALCL), Sézary syndrome (SS), folliculotropic mycosis fungoides (MF), and early‐stage MF.^13^, ^14^ Seven of 9 (78%) of recurrent LPDs occurred after the Pfizer‐BioNTech vaccine, and 2 of 9 (22%) occurred after the Oxford‐AstraZeneca vaccine. Seven of 9 relapses (78%) occurred in men, with 2 relapses in women (22%). The mean age was 62 years (range 47–79). Six recurrences were reported in Italy, two in Greece, and one in the US. Recurrences occurred on average 10 days after vaccination (range 2–15 days).

**FIGURE 1:**
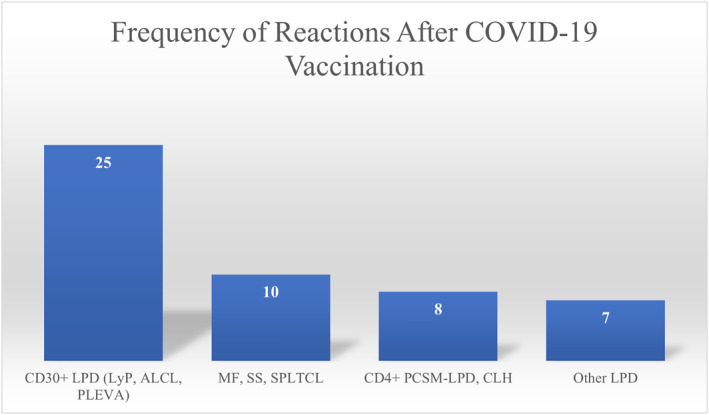
Number of LPDs by type after COVID‐19 vaccination. LyP, lymphomatoid papulosis; ALCL, anaplastic large cell lymphoma; PLEVA, pityriasis lichenoides et varioliformis acuta; MF, mycosis fungoides; SS, Sézary syndrome; SPLTCL, Subcutaneous panniculitis‐like T‐cell lymphoma; PCSM‐LPD, primary cutaneous CD4+ small/medium sized pleomorphic T‐cell lymphoproliferative disorder; CLH, cutaneous lymphoid hyperplasia; LPD, lymphoproliferative disorder.

Combined, there were 25 cases of CD30+ LyP (14/50, 28%), PLEVA (7/50, 14%), and ALCL (4/50, 8%), 10 of MF (5/50, 10%), SS (3/50, 6%), SPTCL (2/50, 4%), 8 of CLH (4/50, 8%) and CD4+ PC‐SMPTCL (4/50, 8%), and 7 others (Figure 1).

### 3.2 Pfizer‐BioNTech (mRNA vaccine)

The Pfizer‐BioNTech vaccine was associated with LPDs in 36/50 (72%) cases. We found that 21 of 36 (58%) Pfizer‐BioNTech vaccine reactions occurred in men, with 15 occurring in women (42%). The mean age of reaction to Pfizer‐BioNTech was 47 years old (range 15–80). Eighteen of 36 (50%) Pfizer‐BioNTech reactions were reported from the United States (US), with 15 from Italy (42%), and single cases from Canada, Greece, and Romania. Eleven of 36 reactions were LyP (31%), with three ALCL or CD30+ LPDs (8%). Seven reported reactions were MF or SS, five were CD4+ PSCM‐LPD, four were PLEVA, and four were CLH. Sixteen of 36 (54%) reactions occurred after the first dose of the vaccine, with 20 reactions occurring after subsequent doses. Reactions occurred on average 14 days (range 2–42 days) after Pfizer‐BioNTech vaccination. Twenty‐eight of 32 (88%) reports which included evaluation for CD30 status reported CD30 positivity.

### 3.3 Moderna (mRNA vaccine)

The Moderna vaccine was associated with seven LPD reactions (7/50, 14%). Six of 7 (86%) reactions occurred in men, with only one reaction reported in a woman. The average age was 53 years (range 22–79 years). Five reactions were reported from the US, one from Japan and one from Spain. Two patients developed PLEVA, one LyP, one ALCL, one non‐specific lymphomatoid reaction, one primary cutaneous gamma/delta T‐cell lymphoma, and one SPTCL. Two of 7 reactions occurred after the first dose, with the rest presenting after the second or third doses of the vaccine. Reactions occurred on average 6 days (range 3–10 days) after Moderna vaccination. Three of five cases (60%) with reported staining detected CD30 positivity.

### 3.4 Oxford‐AstraZeneca (adenovirus‐vectored vaccine)

Oxford‐AstraZeneca was associated with six reactions (6/50, 12%). Three reactions occurred in men and three in women. The average age was 65 years (range of 56–79 years). Three reactions were documented in Greece, with two from Italy and one from Colombia. Two reactions of LyP were reported, one ALCL, one PLEVA, 1 MF, and one pcPTCL. Five of 6 (83%) reactions occurred after the first dose of the vaccine. Reactions occurred on average 12 days (range 7–28 days) after Oxford‐AstraZeneca vaccination. Five of 6 (83%) cases demonstrated CD30 positivity.

### 3.5 Johnson & Johnson (adenovirus‐vectored vaccine)

Johnson & Johnson was associated with only one reaction, SPTCL, in a 28‐year‐old woman, after her first dose of the vaccine. The reaction occurred 3 days after vaccination. This case was documented in the US and did not demonstrate CD30 positivity.

### 3.6 Combined analysis

The average age of patients was 53 years, with a slight male predominance (male‐to‐female ratio 1.5:1). Geographical distribution revealed the most reported cases in the United States (24), Italy (17), and Greece (4), with single cases in Spain, Colombia, Canada, Japan, and Romania. Reactions occurred on average 13 days (range 2–42 days) after vaccination Histopathology revealed CD30+ in 80% of cases. The most common LPD was lymphomatoid papulosis (30%) (Figure 1). We did not find a pattern in the anatomic location of lesions or association with vaccine site.

All cases resulted in complete or near‐complete remission of the LPD, regardless of therapy which ranged from observation to steroids, methotrexate, or excision.

## 4 DISCUSSION

We describe LPDs by vaccine manufacturer for 50 cases of LPDs after COVID‐19 vaccination.

We found that 72% of LPDs involved Pfizer‐BioNTech (mRNA), the most administered vaccine (∼70% of market share) internationally. Moderna (mRNA) makes up ∼20% of worldwide vaccinations and 14% of LPDs in our dataset. Oxford‐AstraZeneca (adenovirus‐vectored) comprises ∼7% of global vaccines and 12% of LPDs in our dataset. Its administration was limited due to health concerns such as blood clots. Johnson & Johnson (adenovirus‐vectored) comprises less than 2% of global vaccines and makes up 2% of LPDs in our analysis.

Pfizer‐BioNTech reactions were most documented in the US and Italy; Moderna reactions in the US, Japan, and Spain; Oxford‐AstraZeneca from Greece, Italy, and Colombia; and Johnson & Johnson from the US. Analysis of LPDs by country demonstrated predominance of Pfizer‐BioNTech reactions in the US, Italy, Canada, and Romania, Oxford‐AstraZeneca in Greece and Colombia, and Moderna in Spain and Japan. Pfizer‐BioNTech‐associated LPDs were not reported in Greece (aside from one case), Spain, Colombia, or Japan possibly due to higher rates of other vaccines in these countries or limited cases. Of note, vaccine reactions were predominantly reported from Western countries which used similar vaccine types. Reporting bias likely explains the lack of reports from other countries, although it is possible that the different vaccines used in those countries may be less associated with LPDs. Pfizer‐BioNTech, Moderna, and Oxford‐AstraZeneca comprised similar proportions of reactions in our dataset to their global distribution rates. Thus, LPDs may arise with different vaccine types rather than mRNA vaccines exclusively and appear proportional to global market shares.^4^

Pfizer‐BioNTech has been used in at least 146 countries, Oxford‐AstraZeneca in 141, Johnson & Johnson in 111, and Moderna in 87. The initial efficacy for preventing infection based on pre‐authorisation clinical trials was highest with Moderna and Pfizer‐BioNTech at over 90%. Oxford‐AstraZeneca has an efficacy rate of 74% and Johnson & Johnson of 52%.^15^ One study of cutaneous reactions to COVID‐19 vaccines in Turkey found elevated rates of both local injection site and systemic reactions after the Pfizer‐BioNTech vaccine compared to Sinovac/CoronaVac (inactivated SARS‐CoV‐2). These investigators suggested that the Pfizer‐BioNTech vaccine triggers an inflammatory pathway through polyethylene glycol (PEG), activating the innate immune system, leading to skin‐resident memory T‐cell activation and a Th17/Th22‐predominant environment, causing neutrophil flow and triggering cutaneous reactions.^16^ Thus, not only are vaccines generally proportional to global market shares, but their rates of reaction may also reflect their efficacy in generating robust immune responses. For example, Johnson & Johnson may provoke the lowest rates of LPDs in part because of its low distribution but also due to its low efficacy at stimulating the immune system to produce antibodies.

Pfizer‐BioNTech and Moderna are mRNA vaccines.^[^ ^17^, ^18^, ^19^ ^]^ The Pfizer‐BioNTech vaccine is a lipid nanoparticle‐formulated, N1‐methylpseudouridine (m1Ψ)‐modified mRNA that encodes full‐length transmembrane spike protein with two proline substitutions at residues K986 and V987 to stabilise the protein in perfusion conformation (S‐2P).^[^ ^20^, ^21^ ^]^ Similarly, Moderna contains lipid nanoparticle‐formulated m1Ψ‐modified mRNA encoding the S‐2P antigen.^18^ These mRNA vaccines provide cells with genetic material in the form of nucleoside‐modified mRNA delivered in lipid nanoparticles to encode the SARS‐CoV‐2 spike glycoprotein in order to produce antibodies to fight a COVID‐19 infection.

Oxford‐AstraZeneca is a viral vector vaccine.^22^ Specifically, the vaccine is composed of a recombinant, replication‐deficient chimpanzee adenovirus (ChAdOx1) vector encoding the full‐length wild‐type SARS‐CoV‐2 spike protein with N‐terminal tissue plasminogen activator signal sequence that moves the protein to the cell surface, ultimately making spike protein.^23^ Johnson & Johnson is also a viral vector vaccine which is made of a recombinant, replication‐deficient human adenovirus type 26 vector encoding full‐length spike protein with the wild‐type signal sequence, 2P, and furin cleavage site mutations.^24^ Viral vector vaccines deliver antigen‐coding genes to host cells to produce an immune response, mimicking a natural infection. While they have different mechanisms, both mRNA and viral vector vaccines produce antibodies based on the spike protein to prepare the body for COVID‐19, which may account for their similar rates of reactions.

Moderna showed the greatest rate of male predominant vaccine reactions (86%) followed by Pfizer‐BioNTech (58%). Oxford‐AstraZeneca had an equal male to female ratio, and Johnson & Johnson was only associated with a single reaction in a woman. Overall, most reactions occurred in men, which could be a result of random chance from a small sample size, though some LPDs are more common in men.^25^

Cutaneous lymphomas are uncommon in the general population. For instance, the estimated incidence of CTCL is 8.55 per million.^26^ LPDs after COVID‐19 vaccination are even less common and associated with distinct age and gender distributions. However, clinical presentation in terms of lesion type and anatomic location are similar for LPDs following and not following vaccination. Most (82%) cases were new LPDs in healthy patients, however, 9 cases (18%) were recurrences of LPDs previously in complete remission. Both new and relapsed LPDs demonstrated Pfizer‐BioNTech and male predominance, however, new LPDs had a younger average age on average. Reactions to Oxford‐AstraZeneca were more common in older patients (average 65 years), while with Pfizer‐BioNTech reactions were identified in a much younger average age (47 years). The average age at presentation of cutaneous LPDs is 65 compared to 53 for LPDs after COVID‐19 vaccination.^27^ Since vaccine administration to children was delayed, the true mean age may be lower. The difference in average age for new versus recurrent LPDs and for different vaccine types may speak to the immunogenicity of the vaccine. Since older patients are more likely to already have LPDs, it is more likely that older patients would have recurrences compared to younger patients. For example, there may be higher rates of reaction to Oxford‐AstraZeneca in older patients because this vaccine stimulates a less rigorous immune response, thus producing reactions in patients who tend to be older and more susceptible to antigenic reactions.

Other than for Moderna, most vaccines were associated with reactions occurring after the first dose of the vaccine. Given that many patients developed LPDs after their initial dose, a question is raised about previous exposure to the antigen. It is possible that these patients had a previous asymptomatic COVID‐19 infection, resulting in development of memory T‐cells before vaccination. Reactions occurred at a mean of 13 days after vaccination, with fastest onset for Johnson & Johnson followed by Moderna, Oxford‐AstraZeneca, and Pfizer‐BioNTech. Johnson & Johnson and Moderna may have relatively faster LPD onset due to small sample size or possible underlying predisposition in those individuals. Conversely, Pfizer‐BioNTech may be associated with more insidious onset of LPDs. The temporal relationship between vaccination and LPDs development may suggest a potential relationship.

Eighty percent of cases demonstrated CD30 positivity. In the case of Pfizer‐BioNTech, 88% of cases demonstrated CD30 positivity, with only 60% in Moderna, 83% in Oxford‐AstraZeneca, and none in Johnson & Johnson. CD30 is a tumour necrosis factor (TNF) superfamily receptor expressed on activated T‐cells, which can exhibit acquisition of cytotoxic proteins, granzyme or TIA, contributing to a strong immune response. CD30 can be expressed in various conditions, induced by antigens, mitogens, arthropod bites, or viruses.^28^ In the event of CD30 antigenic activation, signal mediation takes place, preventing apoptosis and promoting abnormal cell proliferation. The vaccine spike glycoprotein leads to a powerful adaptive T‐cell and B‐cell immune response including neutralising antibody production. The COVID‐19 vaccine may induce hypersensitivity to components of the vaccine vehicle, adaptive cellular immune responses to human‐manufactured spike glycoprotein, or inherently immune‐enhancing effects.^29^ The COVID‐19 vaccine’s spike protein may serve as an antigenic trigger resulting in clonal expansion of antigenically responsive T‐cells and subsequent oncogenic hits eventuating in CD30+ LPDs.^30^ The different rates of CD30 positivity by vaccine type may reflect limited sample or it may indicate the increased immunogenicity of certain vaccines such as Pfizer‐BioNTech, which may have an especially reactive vaccine component. Of note, there are few reports of LPDs after COVID‐19 infection, further suggesting that a component of the vaccine may be related to these indolent disorders.

The combination of CD30+ LyP, PLEVA (only 1/7 PLEVA cases was CD30 negative), and ALCL comprised 25 cases, with MF, SS, and SPTCL comprising 10, and CLH and CD4+ PC‐SMPTCL comprising 8 (with 7 other LPDs). While PLEVA may not classically be grouped with these other LPDs, intraepithelial atypical lymphocytes, phenotypic abnormalities, and TCR‐gamma rearrangements have led to its consideration as a T‐cell dyscrasia and highlight their similarity, thus we have included it in our analysis.^31^ LyP and ALCL were especially common after the Pfizer‐BioNTech and Oxford‐AstraZeneca vaccine, with PLEVA being most common after Moderna and SPTCL after Johnson & Johnson. While the predominance of CD30+ LPDs following closely after COVID‐19 vaccination, is striking, the connection is not proven. The few cases without CD30 positivity may have occurred through another pathway of T‐cell activation through antigenic stimulation, clonal expansion, and LPD development.

Limitations of this study include limited sample size, reporting bias, publication bias based on language or country of origin, and inability to prove causation. We counted a reaction as CD30 positive if the report labelled it as “CD30 positive” or mentioned significant degree of CD30 positivity, however, we recognise that there is variability in degree of CD30 positivity, which could not be determined by this study.

Importantly, all patients had complete or near‐complete remission of LPDs with treatment ranging from observation to steroids, methotrexate, or excision, showing that LPDs after COVID‐19 vaccination have favourable outcomes. This is consistent with other cutaneous COVID‐19 vaccine reactions, which are also self‐limited and generally short‐lived.^32^ We demonstrate a potential association, not causation, for self‐limited disorders following COVID vaccination. The COVID‐19 vaccine should be administered according to national and international guidelines. Vaccination is recommended to decrease the risk of developing severe COVID‐19 infection, and potential complications.

## 5 CONCLUSION

We investigate different cutaneous LPDs after COVID‐19 vaccination and their association with various vaccine types. Reactions occur proportionally to global distribution, with most LPDs linked to Pfizer‐BioNTech, followed by Moderna and Oxford‐AstraZeneca. These LPDs overall affected younger patients and had male predominance. Most cases had CD30+ positivity, suggestive of antigenic activation in these cases. COVID‐19 vaccination remains safe and important to prevent severe COVID‐19 and complications. LPDs that occurred following vaccination appear to be rare, reactive, self‐limited disorders.

## CONFLICT OF INTEREST STATEMENT

LJG has served as an investigator for and/or received research support from Helsinn Group, J&J, Mallinckrodt, Kyowa Kirin, Soligenix, Innate, Merck, BMS, and Stratpharma; on the speakers’ bureau for Helsinn Group and J&J; and on the scientific advisory board for Helsinn Group, J&J, Mallinckrodt, Sanofi, Regeneron, and Kyowa Kirin. ERG, BDK, CMS, LMF, OA, DQ, MHT, CMM have no conflicts of interest to declare.

## AUTHOR CONTRIBUTIONS

**Emily R. Gordon**: Conceptualisation (equal); Data curation (lead); Formal analysis (lead); Investigation (lead). **Oluwaseyi Adeuyan**: Conceptualisation (supporting); Formal analysis (supporting). **Bradley D. Kwinta**: Conceptualisation (equal); Data curation (equal); Formal analysis (equal). **Celine M. Schreidah**: Conceptualisation (supporting); Formal analysis (supporting). **Lauren M. Fahmy**: Conceptualisation (supporting); Formal analysis (supporting). **Dawn Queen**: Conceptualisation (supporting); Formal analysis (supporting); Methodology (supporting). **Megan H. Trager**: Conceptualisation (supporting); Data curation (supporting); Formal analysis (supporting). **Cynthia M. Magro**: Conceptualisation (supporting); Data curation (supporting); Formal analysis (supporting); Investigation (supporting); Methodology (supporting). **Larisa J. Geskin**: Conceptualisation (equal); Data curation (equal); Formal analysis (equal); Investigation (equal); Methodology (equal).

## ETHICS STATEMENT

Not applicable.

## Data Availability

The data underlying this article are available in the article.
